# Predicting Evolution of the Transcription Regulatory Network in a Bacteriophage

**DOI:** 10.1093/gbe/evy191

**Published:** 2018-09-01

**Authors:** Daniel J Garry, Adam J Meyer, Jared W Ellefson, James J Bull, Andrew D Ellington

**Affiliations:** 1 Center for Systems and Synthetic Biology, Department of Molecular Biosciences, Institute for Cellular and Molecular Biology, University of Texas, Austin; 2 Synthetic Biology Center, Department of Biological Engineering, Massachusetts Institute of Technology; 3 Center for Computational Biology and Bioinformatics, Department of Integrative Biology, Institute for Cellular and Molecular Biology, University of Texas, Austin

**Keywords:** T7, promoter, evolutionary path, sequence space, prediction

## Abstract

Prediction of evolutionary trajectories has been an elusive goal, requiring a deep knowledge of underlying mechanisms that relate genotype to phenotype plus understanding how phenotype impacts organismal fitness. We tested our ability to predict molecular regulatory evolution in a bacteriophage (T7) whose RNA polymerase (RNAP) was altered to recognize a heterologous promoter differing by three nucleotides from the wild-type promoter. A mutant of wild-type T7 lacking its RNAP gene was passaged on a bacterial strain providing the novel RNAP in trans. Higher fitness rapidly evolved. Predicting the evolutionary trajectory of this adaptation used measured in vitro transcription rates of the novel RNAP on the six promoter sequences capturing all possible one-step pathways between the wild-type and the heterologous promoter sequences. The predictions captured some of the regulatory evolution but failed both in explaining 1) a set of T7 promoters that consistently failed to evolve and 2) some promoter evolution that fell outside the expected one-step pathways. Had a more comprehensive set of transcription assays been undertaken initially, all promoter evolution would have fallen within predicted bounds, but the lack of evolution in some promoters is unresolved. Overall, this study points toward the increasing feasibility of predicting evolution in well-characterized, simple systems.

## Introduction

The historical dichotomy between studying living systems through reductionist molecular biology or holistic evolutionary biology has given way to a unification termed the functional synthesis (Dean and Thornton 2007). This synergistic approach is increasingly used to empirically validate evolutionary hypotheses and has been applied to such disparate subjects as insecticide resistance, mouse coat color, and antibiotic resistance (Newcomb et al. 1997; Hoekstra et al. 2006; Weinreich et al. 2006; Dean and Thornton 2007). A long-term goal of the functional synthesis that may now be within reach is evolutionary prediction of phenotype evolution and the genetic pathways leading to those phenotypes. Predicting organismal moves on a fitness landscape requires systems in which the genotype–phenotype relationship is well characterized, and the fitness consequences of the different phenotypes are also understood. At this time, these properties are most feasibly extracted from systems in which there are relatively few alternative molecular states that nonetheless have major effects on fitness.

A simple, yet still complex, system that seems particularly amenable to evolutionary prediction is the transcriptional regulatory network of bacteriophage T7. Bacteriophage T7 is a lytic, 40-kb dsDNA podovirus that infects *Escherichiacoli* (Molineux 2006). The genome encodes nearly 60 genes and is unusual among phages in that most gene expression requires a phage-encoded RNA polymerase (RNAP). Gene expression is controlled by 17 phage promoters in the genome, and there are a number of overlapping transcripts. However, underlying this complex regulatory network is a single phage RNAP (T7 RNAP) that interacts with a highly defined and well-characterized set of promoters. We and others have previously used protein engineering and directed evolution to craft T7 RNAP variants that recognize alternative promoter sequences (Ellefson et al. 2014; Meyer et al. 2015).

In particular, we have generated a T7 RNAP variant, the G78-KIRV RNAP (Ellefson et al. 2014) that can utilize a promoter variant that is three nucleotides away (CGG) from the consensus wild-type T7 promoter (GAC) at positions -11 to -9 (Rong et al. 1998). (Fifteen of the 17 promoters have GAC in these positions, the other two have AAC.) The regulatory system of this G78-KIRV RNAP with its novel promoters is orthogonal to regulation in the wild-type phage, in that the polymerases cannot readily cross-utilize one another’s promoters. The synthetically derived G78-KIRV polymerase enables us to perturb the regulatory network of T7 phage and observe evolution of the network in response to a major shift in specificity of the transcriptional apparatus. Most importantly, the observed evolutionary pathways for promoters can be compared with initial predictions based on the known and fixed promoter specificities of the mutant G78-KIRV RNAP.

## Materials and Methods

### Phage Passaging

Frozen aliquots of BL21-Gold cells (Agilent) transformed with pLUV-G78-KIRV RNAP (wild-type T7 RNAP with Q744K, L747V, N748H, L749I, R756E, L757M, H772R, and E775V mutations, from Ellefson et al. 2014) were thawed and used to inoculate 10 ml of 2xYT with proper antibiotic and grown at 37 °C to achieve a density of 10^8^ cells/ml at 60 min. For the first 50 passages, isopropyl-l-thio-β-galactoside (IPTG) was added to a concentration of 1 mM before the 60 min of growth (no IPTG addition for the second 50 passages). After 60 min to reach density, 1 µl of previous bacteriophage lysate was added (a 10^−4^ dilution, transferring approximately 10^7^–10^8^ phage). Bacteriophage was allowed to grow under the same conditions, occasionally allowing the cultures to reach full lysis in order to stimulate recombination.

### RNAP Purification

RNAPs were purified via Ni-NTA N-terminal 6xHis methods in a manner similar to elsewhere (Ellefson et al. 2014). Briefly, pQE-WT/G78 RNAP variants (plasmid sequences and maps included in supplementary figs. S6–S10, Supplementary Material online), each respective RNAP under the control of a T5 promoter/Lac operator, were transformed into BL21-Gold cells and grown overnight in 4 ml of 2xYT. The next day, cells were diluted into 50 ml of 2xYT media and grown to a density of OD_600_ ∼0.6–0.7, when they were induced with 1 mM IPTG. These cells were allowed to grow for 14–16 h at 18 °C, at which point they were pelleted by spinning in centrifuge at 4,000 × g and re-suspended in 30 ml of binding buffer (500 mM NaCl, 5 mM imidazole 50 mM Tris pH 8). This was sonicated at 50% amplitude for 2 min (1 s ON, 1 s OFF) in an ice bath. Lysed cells were spun at 10,000 × g for 30 min, then the supernatant was added to a 1-ml Ni-NTA resin column which had been equilibrated with 2× binding buffer. The cells were washed with 6× column volumes of wash buffer (500 mM NaCl, 20 mM imidazole, 50 mM Tris pH 8) then eluted into 3 ml of elution buffer (500 mM NaCl, 250 mM imidazole, Tris pH 8). These samples were dialyzed into 1.2 l of dialysis buffer (300 mM NaCl, 1 mM ethylenediaminetetraacetic acid, 1 mM DTT, 50 mM Tris pH 8) overnight and again into 1.2 l of storage buffer (100 mM NaCl, 1 mM ethylenediaminetetraacetic acid, 1 mM DTT, 50 mM Tris pH 8) overnight. The resulting protein was adjusted to 1 mg/ml and added to equal volumes of glycerol.

### In Vitro Transcription

In vitro transcription was done in a manner similar to elsewhere (Ellefson et al. 2014). In vitro transcription reactions of the spinach aptamer consisted of 40 mM Tris–HCl pH 7.0, 30 mM MgCl_2_, 6 mM spermadine, 6 mM each NTP, 10 mM DTT, 0.5 µM T7 RNAP, 0.5 µM DNA template, and 0.17 mg/ml DFHBI in DMSO. Reactions were incubated at 37 °C and were read every minute at an excitation/emission of 469/501 nm for 2 h in a Safire monochromator (Tecan). DNA templates were made by thermal cycling 2 µM forward primer and reverse primers (see supplementary table S4, Supplementary Material online) with Accuprime Pfx in its buffer using the following, then gel purified: (94 °C: 2 min, 12 cycles [94 °C: 15 s, 50 °C: 30 s, 68 °C: 30 s], 68 °C: 1 min).

### Lysis Curves

Cells were grown in a manner identical to those of the passaging scheme (achieve a density of 10^8^ cells/ml), and 150 µl of cells was added to each well of a clear, 96-well plate. The proper concentration of IPTG (where required) was added to each well, as, too, was the proper concentration of bacteriophage (determined by previous titer) in order to give the required multiplicity of infection (MOI) (MOI for lysis curves here = 0.01). The plate was grown in a PowerWave 340 microplate spectrophotometer (BioTek) at 37 °C and OD_600_ readings were taken every minute, preceded by shaking for 10 s and intensity level 4.

### Preparing Frozen Cell Stocks for Fitness Assay

pLUV-G78-KIRV-RNAP plasmid variants (plasmid sequences and maps included in supplementary figs. S6–S10, Supplementary Material online) were electroporated into BL21-Gold cells and plated overnight. The next day, the lawn was scraped off and put into 10 ml of 2xYT with proper antibiotic and grown for 2 h, after which point 5 ml was put into 300 ml of 2xYT and grown for an additional hour. The cells were then centrifuged for 20 min at 4,000 × g. The 2xYT was decanted and the cell pellets were resuspended in 5 ml of 20% 2xYT glycerol before freezing in −80 °C. Cell samples were withdrawn and concentrations were found which gave and OD600 ∼1.00 after 1 h of growth when added to 10 ml of 2xYT with proper antibiotic (correlating to 10^8^ cells/ml). All fitness assays were done using the same frozen stocks.

### Fitness Assays

Less than 50 fresh plaque forming units (passaged and titered within last 4 days) were added to 4 ml of top agar, 350 µl of bacteria grown for 1 h to an OD600 ∼1.00 (a density of 10^8^ cells/ml), and 50 mg/ml kanamycin before plating on Kanamycin plates. Plates were incubated for 3 h, top agar was removed with spatula and added to 15-ml falcon tubes, to which 4 ml of 2xYT and 1 ml chloroform were added and vortexed, then allowed to sit at 4 °C for at least 1 h. The tubes were then spun at 4,000 × g for 15 min at 4 °C. The aqueous layer after spinning was 5 ml. The phage titer of the aqueous phase was then measured.

### Sequencing and Sequence Analysis

Miseq 2x250 paired-end reads were taken using purified bacteriophage DNA and aligned to the T7 bacteriophage genome (GenBank V01146.1) using breseq (Deatherage and Barrick 2014). At least 3.6 × 10^5^ reads were taken for each sample. The breseq output was then analyzed to identify promoter changes.

## Results

### Initial Prediction of a Single Mutation Path to a Mutant Promoter Triplet

The G78-KIRV RNAP was a product of directed evolution; presumably T7 had never experienced such a polymerase specificity in its evolutionary history. Given the novelty of the G78-KIRV RNAP to T7 and the fact that its promoters were three mutations away from T7 wild type, it was unclear to what extent the native bacteriophage T7 genome would be able to propagate on and evolve if forced to use this polymerase. By forcing T7 to use this new RNAP in place if its own, the phage was confronted with a potentially deep fitness valley that required the acquisition of several mutations to regain robust replication.

**Fig. 1.**
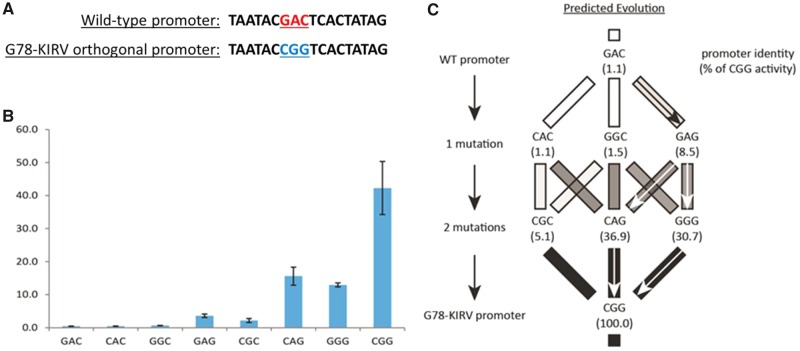
—Promoters and single-step prediction matrix from wild-type to G78-KIRV promoter. (*A*) Wild-type (red) and G78-KIRV (blue) T7 RNAP promoters. (*B*) In vitro activity of the G78-KIRV RNAP on wild-type (GAC), G78-KIRV (CGG), and intermediate promoters. The G78-KIRV RNAP was purified and used for in vitro transcription using a linear dsDNA spinach template with each of the respective promoters (supplementary fig. 13, Supplementary Material online). Fluorescence after 1 h was taken in triplicate shown with standard error as a percentage of wild-type T7 RNAP activity on its cognate promoter (GAC). (*C*) Single-step prediction matrix shows the six intermediate promoters as they take single steps from the wild-type promoter to the G78-KIRV promoter. The boxes are shaded relative to each respective promoter activity using the G78-KIRV RNAP, and arrows show the hypothesized most likely steps through mutational space based on in vitro transcription activity assay. Percentage activity of the G78-KIRV RNAP on the CGG promoter is shown in parentheses.

To determine the most likely single-step evolutionary paths for the bacteriophage promoter network utilizing the G78-KIRV RNAP, we carried out in vitro transcription assays using purified G78-KIRV RNAP on the wild-type T7 promoter (GAC), on the CGG G78-KIRV promoter, as well as on the three single- and three double-mutant promoters intermediate between the wild-type GAC and the G78-KIRV CGG promoters (fig. 1). These transcription reactions confirmed the previously observed orthogonality (lack of activity of heterologous promoter–RNAP combinations) but, more importantly revealed the transcriptional activity of the six intermediate mutant promoters (fig. 1). Consistent with our a priori expectations of how evolution would proceed through single-step intermediates, the G78-KIRV polymerase had a higher activity on all six intermediate mutant promoters than on the T7 wild-type triplet (GAC). Of the three promoters, one mutation from wild-type, the GAG promoter displayed the highest activity and was therefore regarded as the likely first step of evolution. Of the three double mutants (one mutation away from the G78-KIRV triplet, CGG), both the CAG and GGG promoters exhibited substantially higher transcriptional activity than CGC and also higher activity than any of the single-step mutants (fig. 1). These results thus provided a foundation for predicting the evolution of T7 RNAP promoters in the phage challenged with the G78-KIRV polymerase.

There are 64 triplets that constitute the full genotype space for evolution in this system (neglecting promoter duplications and deletions within the promoter sequence itself). Our characterization of just eight triplets (six intermediates) stemmed from a combination of pragmatism and the prediction that evolution would follow direct, single-step sequence changes between the two parental triplets. Validating and characterizing even a single promoter is laborious, so limiting the study to one-eighth of the comprehensive set was effectively necessary.

### T7 Evolves to Use the Alternative Polymerase

In initiating the evolution of wild-type T7 in response to the new RNAP (the G78-KIRV RNAP), we first determined whether a phage variant whose genome lacked the wild-type RNAP gene (T7Δ1) could grow with the G78-KIRV polymerase expressed in trans from a plasmid. The deletion in T7Δ1 removes not only the RNAP gene but also the first T7 promoter (φ1.1A); for the purposes of our study, we attribute no significance to this missing promoter as φ1.1B—still present—lies within 100 bases of φ1.1 A and there are no intervening genes. T7Δ1 was complemented by the plasmid, but its plaques were noticeably smaller than plaques of wild-type T7 (supplementary fig. S1, Supplementary Material online), suggesting a fitness deficiency. The fact that plaques could be generated at all was surprising given the three-mutation distance between the optimal triplet for the G78-KIRV RNAP and the promoters found in T7 wild-type. It seems likely that the overexpression of the G78-KIRV polymerase before phage infection is at least partially responsible for the complementation. (Indeed, a wild-type T7 with the RNAP gene replaced with the G78-KIRV polymerase did not form plaques; Daniel J. Garry, unpublished results.) The reduced initial growth meant that the phage could be expected to evolve higher fitness after serial passaging.

**Fig. 2.**
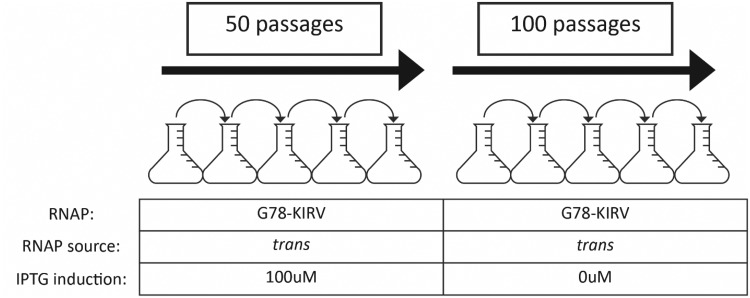
—Passaging scheme over course of experiment. Four replicate T7Δ1 phage lines were initially subjected to 50 passages on BL21 cells expressing the G78-KIRV RNAP mutant using 100 µM IPTG in trans (i.e., from a plasmid). Passaging was continued for another 50 passages in the same manner using 0 µM IPTG induction.

Four replicate lines of T7Δ1 were subjected to repeated serial transfer on the complementing host (fig. 2). By providing the RNAP in trans, all regulatory evolution was forced to occur in the promoters. BL21 *E. coli* cells harboring the G78-KIRV RNAP expression plasmid were grown for 1 h with 100 µM IPTG induction, at which point T7Δ1 phage was added at an MOI of 0.01–0.1 and the culture grown until lysis. This protocol allowed expression of the complementing RNAP in all cells prior to phage infection. The parental T7Δ1 lysed the culture within ∼90 min, whereas either wild-type T7 phage or T7Δ1 with the wild-type polymerase provided in trans fully lysed the culture in ∼30 min. Over the course of the first 50 passages the lysis time decreased, ultimately going from 90 to 30 min in the presence of IPTG induction (supplementary fig. S2, Supplementary Material online). At this point, serial passaging was continued without IPTG induction, relying only on the leakiness of the LacUV5 promoter to drive expression of the G78-KIRV RNAP. Omission of IPTG induction resulted in an initial increase in culture lysis time to ∼60 min, which then again decreased back to ∼30 min during passages 51–100 (supplementary fig. S3, Supplementary Material online).

**Fig. 3.**
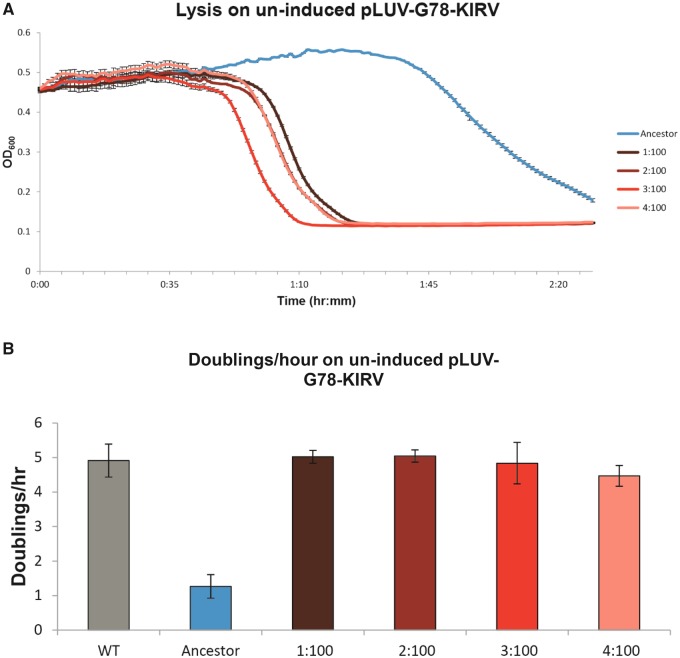
—Lysis time and fitness assays after 100 passages. (*A*) Whole culture lysis curves (MOI = 0.01) for each of the four evolved strains after 100 passages (∼72 h of adaptation) relative to the original T7Δ1 ancestor. (*B*) Plate growth fitness assays (calculated as doublings/hour) comparing WT T7, T7Δ1, and the four evolved strains after 100 passages. The WT T7 phage carries its own RNAP, so the complementation provided by these hosts is not necessary. Note that these assays were conducted from the increase in titer during 3-h growth on plates; the growth rate under these conditions is far less than in liquid (Bull et al. 2011).

After 100 passages, the culture lysis time (measured on uninduced G78-KIRV RNAP cells) was dramatically shorter in all four replicates than with wild-type T7 (fig. 3*A*). Because time to lyse a culture is an indirect fitness measure, fitness assays of growth on plates were performed in which phage was allowed to grow for a fixed time on uninduced G78-KIRV RNAP cells. Fitness assays were carried out for the wild-type T7 phage, the ancestor T7Δ1 phage, and the four evolved T7Δ1 phage lines at passage 100. These fitness assays showed that the four evolved strains reached nearly the same doublings per hour as wild-type T7 phage on uninduced G78-KIRV RNAP cells (fig. 3*B*). We therefore set out to identify the mutations for increased fitness, focusing on the network of T7 promoters.

### Limited Promoter Changes and Promoters That Changed in the Evolved Lines

The populations of all four adapted lines were sequenced at two time points, after transfer 50 and after transfer 100 (utilizing MiSeq 2x250 reads with at least 3.6 × 10^5^ reads per sample). We highlight several points before describing details.

**Fig. 4.**
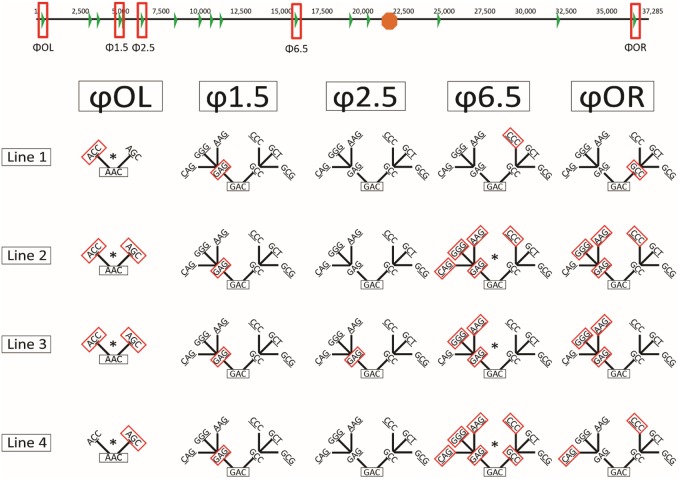
—Promoters mutated from the wild-type T7 promoter in the course of the experiment. (Top) Map of the T7Δ1 genome with the 16 T7Δ1 promoter locations as green triangles and the T7 terminator as orange octagon. The five promoters evolved during the course of the passaging are shown in red boxes. (Below) The evolution of different promoters (passage 100) is shown in red boxes on “trees” depicting single-step mutation options for the five promoters that evolved. The bottom of the tree is the triplet in the T7 wild-type promoter (not all T7 promoters are the same). The tree extends one to three possible mutational steps. Duplications are identified with an asterisk (*).

1. Across the four lines, substitutions occurred in only 5 of the 16 T7Δ1 promoters: φOL, φ1.5, φ2.5, φ6.5, and φOR (fig. 4). As the triplet in φOL is AAC, we first address the nature of evolution in the other four promoters, all of which have the GAC triplet. Figure 4 includes the evolution of φOL, and the text addresses φOL in a dedicated paragraph below.
2. No promoter evolved to the expected CGG triplet. Furthermore, no promoter evolved changes at more than two bases.
3. Two single-step mutants were observed. One (GAG) was the predicted first step; the other (GCC) lay outside the six intermediates on direct paths between the two parental triplets.
4. As expected, the two single-step intermediates with low G78-KIRV activity did not evolve.

**Fig. 5.**
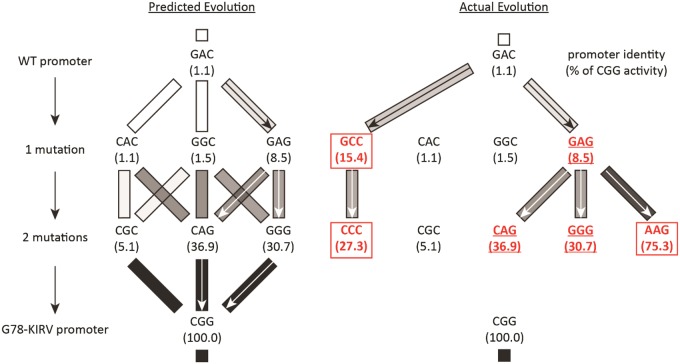
—Predicted versus actual promoter changes in the passaging. Predicted (on left, as before) and actual mutations (on right) found during the passaging. Actual paths (red promoters) on right are shown with arrows, and pathway boxes are shaded according to activity. Activity as a percentage of the G78-KIRV T7 RNAP on the CGG promoter is displayed in parentheses below each triplet (fig. 1 and supplementary fig. S7, Supplementary Material online). Those promoters found that matched the prediction are underlined whereas those unanticipated by the prediction are boxed.

In the initial 50 passages, all four lines exhibited an anticipated GAG single change toward the promoter specificity of the G78-KIRV RNAP in one to three of the promoters (supplementary table S1, Supplementary Material online). But unanticipated CCC and GCC changes were also observed in all four lines in one to three of the promoters (supplementary table S1, Supplementary Material online). By passage 100, promoters were found with two substitutions toward the G78-KIRV triplet (fig. 4). But yet another unanticipated change (AAG) was found in one to two promoters in three lines (supplementary table S1, Supplementary Material online, and fig. 5).

Individual promoters followed different paths. Most promoters failed to evolve at all, but even those that did evolve often failed to carry the same substitutions as others that evolved. Evolution of the φ2.5 promoter was observed in only one line (in line 3 to GAG), and the mutation did not fix in the population, occurring in 46.6% of the phage by passage 50 but only 25.6% by passage 100 (fig. 4 and supplementary table S2, Supplementary Material online). In contrast, the φ1.5 promoter evolved to GAG all four populations by passage 50 and remained fixed through passage 100. These two promoters can be viewed as the limit cases for others; in one case, virtually no evolution occurred (the φ2.5 promoter evolution in a fraction of line 3), but in another, there was rapid evolution toward a single endpoint, suggesting strong selection for a particular sequence (the rapid fixation in all lines at the φ1.5 promoter).

The φOR promoter first evolved to GCC in all four lines by passage 50. It then proceeded to evolve into a heterogeneous mixture of GCC, GGG, AAG, GAG, and CCC by passage 100 (fig. 4).

Interestingly, the noncanonical φOL promoter, whose triplet is AAC instead of GAC, followed a different path, evolving to AGC in 39% of the line 4 population by passage 50 (supplementary table S2, Supplementary Material online) (Dunn and Studier 1983). Our activity assays did not test triplet AAC nor some of its mutants on a direct path to CGG, so there was little basis for attempting predictions of this promoter. The fact that this promoter evolved is interesting because it is considered to be used only weakly. By passage 100, the φOL promoter in lines 1, 2, and 3 evolved to ACC and additionally to AGC in line 3 (fig. 4 and supplementary table S1, Supplementary Material online). Unexpectedly, the AGC substitution in 39% of the line 4 population at passage 50 decreased to only 3.2% of the population by passage 100 (supplementary table S2, Supplementary Material online). This coincided with and may possibly be explained by widespread duplication of the φOL promoter in all lines by passage 100, including duplications with mutant promoters (fig. 4 and supplementary table S2, Supplementary Material online).

The φ6.5 promoter seemed to follow a combination of these paths. It was extremely heterogeneous at both passage 50 (GAC, GAG, CCC, and GCC) and 100 (GAG, CCC, GCC, GGG, and AAG), and also eventually duplicated in lines 2, 3, and 4 (supplementary tables S1 and S2, Supplementary Material online).

### Evolution Outside of Promoters

Following 50 passages, each of the four lines had fixed between two and seven non-promoter substitutions (supplementary table S3, Supplementary Material online). Following 100 passages, the four lines had fixed between one and six non-promoter substitutions (supplementary table S3, Supplementary Material online). Interestingly, the lines after 100 passages retained only a fraction of the fixed substitutions observed at 50 passages (with one line losing all of the fixed, non-promoter mutations, two lines retaining one mutation, and one line retaining two mutations). The fact that most molecular evolution occurred within promoters is consistent with the expectation that most selection operated on polymerase–promoter binding. Of note, the only fixed non-promoter mutation found in all of the lines was an E375K mutation in the minor capsid protein, a change commonly seen in T7 adaptations.

### In Vitro *Transcription of Unanticipated Promoter Triplets*

The observed triplets GCC, CCC, and AAG had not been tested for in vitro activity because they were not on the direct evolutionary pathway between the wild-type and the G78-KIRV RNAP promoter sequences. After discovering these triplets in the evolved phages, purified wild-type and G78-KIRV RNAPs were tested for their activities on these new promoters (fig. 5 and supplementary fig. S4, Supplementary Material online). In line with the serial passaging results, the G78-KIRV RNAP was as or more active on these three unanticipated promoters than on the three predicted single-step mutants (supplementary figs. S1 and S4, Supplementary Material online).

## Discussion

This study is one of still few examples in which the genetic landscape of a system component has been used to perturb and predict the evolution of an interacting system as a whole, in this case the bacteriophage T7 RNAP and the bacteriophage T7, respectively. T7 deleted of its own RNAP gene (T7Δ1) was forced to use a laboratory-evolved variant of T7 RNAP (the G78-KIRV RNAP). This G78-KIRV RNAP variant only weakly transcribed wild-type T7 promoters, and its optimal promoter sequence was three bases removed from the consensus T7 wild-type promoter. Growth of T7Δ1 in this environment was expected to favor evolution of possibly all T7 promoters to match the optimum for the G78-KIRV RNAP.

At the outset, we formed a prediction matrix (fig. 1) for the most likely evolutionary path in response to the novel transcription machinery. This prediction was based on the hypothesis that sequence evolution would follow direct, one-step paths between the parental promoters. T7Δ1 has 16 promoters that could potentially evolve, and we expected that all might evolve in response to the selection, at least when combining results across all replicates. Yet mutations evolved in only 4–5 promoters, and there was a high degree of parallelism across replicates in which promoters evolved. Interestingly, these were the same promoters that evolved in a previous study in which T7Δ1 was forced to use T3 RNAP (Bull et al. 2007). Use of T3 RNAP requires only a single mutational change in a promoter to achieve high transcription activity. The similarity between both studies in identities of promoters that evolved, and equally in so few promoters evolving, suggests that these promoters are the primary transcriptional hubs that are under the highest selective pressure. It should be noted, however, that there is a major difference between the normal T7 infection cycle and the one that operated here. In the present study, the RNAP was present in the cell prior to infection. In the normal life cycle, T7 RNAP is expressed only after phage infection, and then only at low levels. This difference may greatly affect which promoters are important for proper timing of gene expression.

The spacing of the mutant promoters suggests that the inherent processivity of the T7 RNAP is high enough to result in sufficient gene expression of essential phage genes along the T7 genome. The T7 genome has two known terminators, one for *E. coli* RNAP at position 7555 and one for T7 RNAP immediately after gene *10* at position 24170. Transcription from the three *E. coli* promoters in the initial 750 bp of the phage genome by the *E. coli* RNAP produces transcripts of up to 7 kb (Molineux 2006). The φ1.5 and φ2.5 phage promoters, which start at nucleotides 7761 and 9090, respectively, are ∼10 kb away from the φ6.5 promoter, and in the absence of downstream promoters, the φ6.5 promoter would need to transcribe ∼20 kb to produce all of the necessary late genes. While transcripts from these promoters alone would be quite long, T7 RNAP has the ability to transcribe up to 27 kb in vitro and up to 32 kb in vivo (Schelle and Thiel 2002; Mairhofer et al. 2013). Alternatively, the G78-KIRV RNAP may still have enough activity on the remaining wild-type promoters to enable limited transcription.

The evolutionary paths chosen made sense in terms of the in vitro transcription activities of the G78-KIRV RNAP on alternative promoter sequences, but only when transcription was evaluated on a larger set of promoter sequences than lie on direct mutational paths between the wild-type T7 promoter and the optimal G78-KIRV promoter (fig. 5). Some of the promoters that evolved matched the expected single- and double-mutant paths. Some did not, but the mutations that accumulated revealed highly active promoter variants outside the direct mutational pathways between the parental promoters (fig. 5 andsupplementary fig. S4, Supplementary Material online). The data from all in vitro transcription experiments were surprisingly predictive of the evolutionary paths taken by the phage in vivo, so the failure of our initial predictions lay chiefly in our failure to anticipate a broad enough set of promoter sequences. Single-mutant paths at passage 50 had, by passage 100, continued to accumulate functional mutations (e.g., φ6.5 in line 3; supplementary table S1, Supplementary Material online). Some paths seen in one line at passage 50 were reproduced in other lines by passage 100 (e.g., φOR in lines 2, 3, and 4; supplementary table S1, Supplementary Material online). As another failure of our predictions, however, no promoter in any of the 4 replicate lines evolved the triplet on which the G78-KIRV RNAP was evolved.

Even with these limited results, 1) most promoters did not evolve, and 2) many triplets that experienced evolution could, with one additional mutation, have evolved yet higher activity but did not, despite a long opportunity for evolution. Thus, several known one-step paths to higher activity were not taken. Our analysis of promoter activity did not include all 64 possible triplets, so it is unknown whether other “expected” paths also exist in the genotype space, but knowing that some other high fitness paths exist would only confirm that the system failed to evolve higher activity in many promoters when they were each one step away.

In addition to changes in promoter identities, promoters in all four lines experienced duplication events (fig. 4). Most occurred after transfer 50, under conditions of low expression of the mutant RNAP (supplementary table S2, Supplementary Material online), and thus represent a different solution than point mutations to the problem of how to regain adequate transcription activity. All four lines duplicated and mutated their φOL promoter, presumably supporting the function of the φOL promoter to direct T7 RNAP-mediated genome entry into the host cell (Garcia and Molineux 1995). Similarly, the *E. coli* A2 promoter near the φOL promoter at the 5′ end of the phage genome duplicated in experimental line 4 (supplementary table S2, Supplementary Material online).

Empirical recapitulations of mutational pathways such as those demonstrated in this work can yield insights to how enzymes and organisms traverse fitness landscapes (Poelwijk et al. 2007). As but one example, the TEM-1 β-lactamase enzyme that is known to have high activity toward ampicillin can also evolve the ability to degrade a structurally different lactam, cefotaxime, via five mutations (Weinreich et al. 2006). Assaying fitness for all possible combinations of these five mutations revealed that 18 of the 120 possible mutational pathways resulted in stepwise, monotonic fitness increases toward cephalosporinase activity, with ten pathways being most feasible. This result mirrors our own, in which only a fraction of feasible single-step paths was taken (supplementary fig. S5, Supplementary Material online). Similarly, other work on the recapitulation of mutational steps in higher-order regulatory networks, such as hormone receptor:binding sites and repressor:operator interactions, have reinforced the notion that multiple different single-step evolutionary paths lead to diverse movements on fitness landscapes (Lehming et al. 1990; Bridgham et al. 2006).

Assuming that the evolution results speak to natural selection, extensive promoter heterogeneity and duplication in wild phage should be observed. Indeed, single-subunit T7-like RNAPs with wildly different promoter sequences have been identified not just in other bacteriophages but also in eukaryotic mitochondria and plant chloroplasts (Cermakian et al. 1997; Bohne et al. 2016), consistent with regulatory fitness landscapes that are only moderately rugged, and that conjoin multiple optima within easy reach of one another.

These studies of enzyme evolution and regulatory interaction are useful in displaying critical features of evolution, and they thus lead us to the prospect of predicting the evolution of systems. This prediction of evolutionary paths is what we present here. Our choice to use a heterologous T7 RNAP to evolve T7Δ1 phage was due to the fact that the system is well characterized down to the promoter bases involved in polymerase recognition, that different RNAPs were available, and that the phage is easily evolved (Rong et al. 1998; Bull et al. 2007; Ellefson et al. 2014). Overall, it has been possible to predict some of the evolutionary trajectory of a bacteriophage in response to a synthetically created component, and our work thus points toward the next step in the functional synthesis: a priori predicting rather than post hoc confirmation.

## Supplementary Material

Supplementary Data
